# Key determinants for lowering the risk of joint replacement in weight-bearing joints: a population-based cohort study

**DOI:** 10.1186/s42836-026-00403-9

**Published:** 2026-06-08

**Authors:** Aminu Suleiman, Mojtaba Lotfaliany, Lana J. Williams, Amanda L. Stuart, Richard Page, Stephen Gill, Anusha P. Budehal, Julie A. Pasco

**Affiliations:** 1 Deakin IMPACT – the Institute for Mental and Physical Health and Clinical Translation, School of Medicine, Deakin University, Geelong, VIC 3220 Australia; 2 Barwon Health, Geelong, VIC 3220 Australia; 3 Barwon Centre for Orthopaedic Research and Education, St John of God Hospital, Geelong, VIC 3220 Australia; 4 School of Public Health and Preventive Medicine, Monash University, Melbourne, VIC 3004 Australia

**Keywords:** Joint replacement, Osteoarthritis, Lifestyle, Body composition, Comorbidities

## Abstract

**Background:**

Hip and knee replacement (arthroplasty) is used to treat advanced joint disease by reducing pain and restoring joint function. This study aimed to identify determinants associated with a lower risk of joint replacement (JR).

**Methods:**

Longitudinal data from the Geelong Osteoporosis Study (GOS) were used. JR was identified via linkage with the Barwon Joint Registry, medical records, and self-report. Anthropometry, body composition, and blood biomarkers were obtained. Demographics, lifestyle, and comorbidities were self-reported. Fractures were identified from radiological reports. Participants with JR before baseline were excluded, leaving 2,882 eligible participants (1,436 men, 1,446 women; ages 20–96 years). A time-dependent Cox regression model with age as the primary time scale and sex as a stratification variable was fitted. Forward stepwise and the least absolute shrinkage and selection operator (LASSO) regression identified relevant predictors.

**Results:**

Over a median follow-up of 16.7 years (IQR: 9.7–23.2), 223 participants (7.7%) underwent JR. Factors with the reduced risk of JR included lower body mass index (HR 0.96, 95% CI, 0.94–0.99), lower spine bone mineral density (BMD) (0.84, 0.77–0.92), lower procollagen type 1 N-terminal propeptide (0.69, 0.50–0.96), non-fallers (0.74, 0.55–0.99) and no history of cancer (0.66, 0.48–0.90). Lower dietary calcium intake (0.74, 0.52–1.04) was associated with the reduced risk of JR in the main models, but this association was attenuated after accounting for supplement use. Compared with low socioeconomic status (SES), participants with medium and high SES had higher JR risk (1.91, 1.04–3.50; 1.88, 1.13–3.15), although the association was weaker in women (interaction: 0.48, 0.25–0.91).

**Conclusion:**

Age-related spine degeneration may explain the link between higher spine BMD and JR. The association between lower dietary calcium intake and reduced JR risk is likely driven by confounding by indication or reverse causality. Lower BMI and lower P1NP levels emerged as potentially modifiable pathways. The absence of falls and no history of cancer were associated with reduced JR risk; however, the cancer association was attenuated in the Fine-Gray analysis when competing mortality was accounted for, and the fall association should be interpreted as observational rather than as evidence of a modifiable pathway. Socioeconomic disparities also highlight potential avenues for reducing JR risk. Overall, these findings suggest that biological, lifestyle, and social determinants may play important roles in shaping long-term JR outcomes.

**Supplementary Information:**

The online version contains supplementary material available at 10.1186/s42836-026-00403-9.

## Background

Joint replacement (JR) is a surgical procedure that replaces a severely damaged or diseased joint with an artificial joint. This procedure is performed mainly for individuals with end-stage osteoarthritis (OA), a degenerative joint disease characterised by the loss of articular cartilage and synovial inflammation that leads to joint stiffness, swelling, pain, and loss of mobility [1]. Approximately 528 million individuals are affected by OA worldwide, predominantly among people above 55 years of age [2]. In Australia, OA is the most prevalent arthritic condition reported, affecting 2.1 million individuals [3]. In 2022, OA contributed to 19% of the total disease burden and 1.2% of all deaths in Australia [3]. Knees and hips are the common joints affected by OA [1], accounting for approximately 9% of the adult population [4]. OA is associated with a substantial economic burden. From 2019 to 2020 in Australia, approximately AUD $3.9 billion was spent managing OA, and AUD $2.3 billion on knee and hip replacement procedures [1]. The burden of OA is expected to rise over time due to the increasing risk of OA among the ageing population.

To date, there is no approved treatment to cure OA. People often use lifestyle modification, such as physical activity, exercise, weight management, medications, topical treatments, and physical and occupational therapy to treat pain related to OA. However, JR is the final symptomatic treatment considered for those with severe symptoms [5]. Therefore, nonsurgical management of OA is needed to decrease the burden of JR [4].

Joint degeneration and subsequent need for JR have been associated with patient-related, disease-specific, and lifestyle factors [6]. For example, age, sex, obesity, physical activity, smoking, and comorbidities have been demonstrated to be risk factors of JR in previous studies [7–9]. However, most of these determinants were identified through clinical samples, utilising patients with severe OA, who are already receiving care, which may limit generalizability due to selection bias and retrospective data collection. A review highlights the need for population-based cohort studies to allow for more robust evaluation of modifiable and non-modifiable risk factors of JR [10]. Though very few population-based studies have been undertaken to identify these determinants, where the participants were recruited from the general population regardless of their clinical status. Longitudinal population-based studies using prospective designs by observing individuals before and after the occurrence of the outcome would enhance statistical power, reduce selection bias, and allow for more robust evaluation of risk and protective factors. Thus, a cohort study offers greater generalisability and provides more realistic estimates of disease burden in the broader population. This study aimed to identify the key determinants for lowering the risk of JR in men and women residing in Australia using data from a population-based cohort study.

## Materials and methods

### Participants

This study included women and men participating in the Geelong Osteoporosis Study (GOS), a population-based cohort study in south-eastern Australia [11]. At the time of recruitment, potential participants were randomly selected from the Commonwealth electoral roll, which served as the sampling frame. To be included in the study, participants had to reside in the Barwon Statistical Division for at least six months. Those who were unable to provide informed written consent were excluded from the study. In total, 1,494 women aged 20 to 94 years were recruited between 1993 and 1997, with a 77% participation rate. Similarly, 1,539 men aged 20 to 96 years were recruited between 2001 and 2006, with a 67% participation rate. Women have been reassessed at two, four, six, eight, ten, and fifteen years post-baseline, while men have been reassessed at five and fifteen years post-baseline. For women, data from baseline, 10-year, and 15-year follow-up assessments were included, while for men, data from baseline, 5-year, and 15-year follow-up assessments were used. A detailed description of the GOS has been published elsewhere [11]. All participants provided written informed consent. The study was approved by the Human Research and Ethics Committee at Barwon Health.

**Fig. 1:**
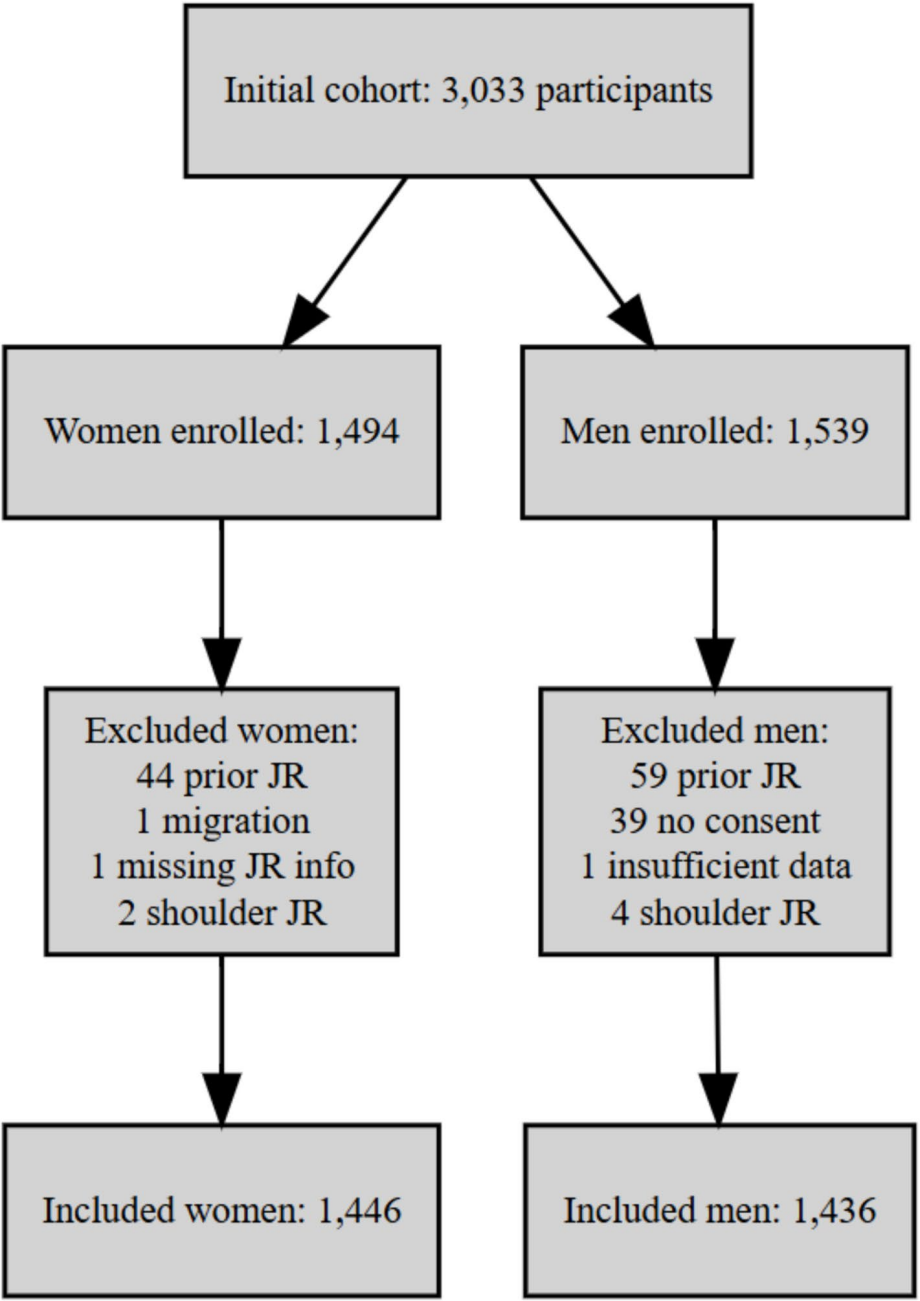
Flow diagram of participant inclusion and exclusion. *Flow diagram of participant inclusion and exclusion. The initial cohort comprised 3,033 participants (1,494 women and 1,539 men). Exclusions were due to prior joint replacement, migration, lack of consent, insufficient data, or shoulder replacement. The final analytic sample included 1,446 women and 1,436 men

For the current study, 44 women were excluded for having JR prior to study commencement, one for non-continued participation due to migration, and one for missing information regarding JR. Similarly, 59 men were excluded because they had a history of JR prior to the study’s commencement, and 39 did not consent to have their medical records examined. One man lacked sufficient data for inclusion. Moreover, due to our focus on weight-bearing joints (hip and knee), two women and four men identified as having shoulder JR were also excluded. Thus, 1,446 women and 1,436 men met the inclusion criteria for this analysis (Fig. 1).

### Measures

#### Joint replacement and mortality

The main outcome in this study, JR, was identified through multiple sources to minimise misclassification of caseness, including data linkage with the Barwon Joint Registry (BJR) [12] and with Medicare records, and confirmed by examination of full-body dual-energy X-ray absorptiometry (DXA) images where possible. The final dataset included hip and knee replacements (which included bilateral procedures and one partial knee replacement). Only the first incident JR per participant was included in the analyses; revision procedures were not considered. We did not differentiate between indications such as OA versus fracture. Mortality data were obtained through data linkage with the Australian Institute of Health and Welfare (National Deaths Index).

#### Socioeconomic status (SES)

The Index of Relative Socioeconomic Disadvantage (IRSD), defined by the Australian Bureau of Statistics (ABS), was used to identify each participant’s socioeconomic status (SES) using socioeconomic indexes for areas (SIEFA) values based on ABS 2011 census data as a level of social disadvantage. SES was expressed in quintiles; one being the most disadvantaged group and 5 the least disadvantaged group [11]. To ensure analytical clarity and sufficient sample size within each group, IRSD was collapsed into three groups, with low as the most disadvantaged group (1st and 2nd quintiles), the medium group (3rd quintile), and high as the least disadvantaged group (4th and 5th quintiles).

#### Clinical measures

Weight was measured in kilograms using electronic scales, and height was measured in metres using a Harpenden stadiometer. Body mass index (BMI) was calculated by dividing weight by height in square metres (kg/m^2^). Waist circumference was measured in centimetres (cm) using an anthropometric tape. Bone mineral density (BMD) of the hip and spine, expressed in grams per square centimetre (g/cm^2^), and whole-body lean mass, expressed in grams, were obtained using DXA (Lunar; Madison, WI, USA). For women, the DPX-L densitometer was used at baseline and at the 10-year follow-up, after which the Prodigy Pro was used at the 15-year follow-up. For some men, BMD was measured using the DPX-L at baseline until the device was replaced by the Prodigy Pro, which was used for subsequent assessments. Cross-calibration between the DPX-L and Prodigy Pro densitometers was performed to ensure measurement consistency across time points and devices. This process involved phantom scanning and regression-based adjustment, minimizing systematic bias and preserving longitudinal comparability [13]. Appendicular lean mass (ALM) was calculated by summing the lean mass of the arms and legs in kilograms and dividing by height in square metres to obtain ALM/h^2^ (kg/m^2^). The BMI and ALM/h^2^ were analysed as continuous variables. Both variables were reverse-coded so that hazard ratios represent the relative risk of JR per 1-unit decrease (BMI per 1 kg/m^2^ decrease; ALM/h^2^ per 1 kg/m^2^ decrease). This coding allowed clearer interpretation of the protective associations of higher BMI and ALM/h^2^ values. Variables were analysed in their original units and were not standardised.

#### Biochemical markers

Blood collection was performed after an overnight fast at baseline and 10-year follow-up for women, and baseline or 5-year follow-up (whichever the first blood sample was collected) and 15-year follow-up for men. Measures of plasma glucose, and serum concentrations of procollagen type I N-terminal propeptide (P1NP), C-terminal telopeptide of type I collagen (CTX), high-sensitivity C-reactive protein (hsCRP), high-density lipoprotein cholesterol (HDL), low-density lipoprotein cholesterol (LDL), total cholesterol (TC), and triglycerides were performed using standardised protocols. The biomarker P1NP, measured in µg/L, was analysed as a continuous variable. To aid interpretation, the variable was reverse-coded so that hazard ratios reflect the relative risk of JR per 1-unit decrease in concentration. This specification emphasises the protective association observed at lower P1NP levels. Analyses were conducted using the original measurement units without standardisation.

#### Lifestyle

Lifestyle factors were documented by questionnaires. Alcohol consumption was categorised as low (never or less than once or twice a week) and high (several times a week or every day). Physical activity was obtained via self-report and categorised as active (very active and active) or non-active (sedentary, limited, inactive, chair or bedridden, and bedfast). Smoking was categorised into three categories: past smoker, current smoker, and non-smoker. Dietary calcium intake was assessed using a dietary intake questionnaire [14]. Dietary intake values, originally recorded in milligrams per day (mg/day), were rescaled to grams per day (g/day) by dividing by 1000 to improve interpretability and analysed as continuous predictors. Continuous variables were reverse-coded so that hazard ratios reflect the relative risk of JR per 1-unit decrease in intake. This specification highlights the protective association observed at lower consumption. Analyses were conducted using the original measurement units without standardisation. All lifestyle measures were collected at each follow-up visit.

#### Comorbidity and medication use

Presence of comorbid conditions and accidents, such as muscle weakness and previous history of falls, was self-reported. Post-baseline fractures were identified from radiological reports, as previously described [15, 16]. Cardio-metabolic conditions, pulmonary conditions, and cancer were defined according to the International Classification of Diseases from the World Health Organisation [17]. Cardio-metabolic conditions included hypertension, hypercholesterolemia, and/or self-reported stroke, transient ischemic attack, and arrhythmias. Hypertension was obtained from measured systolic blood pressure ≥ 140 mmHg and/or diastolic blood pressure ≥ 90 mmHg, and/or medication use (antihypertensive, beta-adrenergic blocking agents, and diuretics) in conjunction with self-report. Hypercholesterolemia was defined as total cholesterol > 5.2 mmol/L, LDL > 3.4 mmol/L, and/or low HDL (< 1.0 mmol/L men, < 1.3 mmol/L women) [18]. Pulmonary conditions included self-reported asthma, bronchitis, or emphysema. Cancer included self-reported lung cancer, bowel cancer, breast cancer, testicular cancer, throat cancer, brain cancer, melanoma, leukaemia, myeloma, or other cancer. Prostate cancer was additionally included for men, and cervical cancer for women. Cancer cases were confirmed by data linkage with the Victorian Cancer Council. All comorbid conditions are referenced against the absence of comorbidities or the lowest-risk group. Likewise, calcium supplement use was recorded at each visit and verified either through the Pharmaceutical Benefits Scheme or by direct inspection of the medication container provided by participants.

### Statistical analysis

Participant baseline characteristics were presented as means and standard deviations for normally distributed variables and as medians and interquartile ranges (IQRs) for non-normally distributed variables. Frequencies and percentages were used for categorical variables. Differences between participants with and without JR were compared using chi-square tests for categorical variables, Student’s t-tests for normally distributed continuous variables, and Mann–Whitney U tests for non-normally distributed continuous variables (where applicable). Predictors of JR were identified using time-dependent Cox regression models, with age as the primary time scale. Covariates were treated as time-dependent in the Cox proportional hazards models. For women, covariates were measured at baseline, 10-year, and 15-year follow-up; for men, covariates were measured at baseline, 5-year, and 15-year follow-up. Each covariate was updated at the time of measurement and carried forward until the next assessment. Time-varying comorbidities were also updated at each wave. Follow-up time was segmented based on the availability of updated covariate information, so that each participant contributed multiple records reflecting their covariate status at different time points. Participants were followed from age at baseline until they had a hip or knee replacement surgery (first surgery), death, loss to follow-up, or study end date (30/05/2023), whichever occurred first. To provide additional context, the models were stratified by joint site (hip vs knee) and by age group (40–59 years vs ≥ 60 years). To ensure that potentially relevant predictors of JR were not missed in the multivariable model, we initially applied forward stepwise Cox regression in an exploratory capacity. To validate the stability of selected predictors and reduce the risk of overfitting, we also performed penalised regression using the least absolute shrinkage and selection operator (LASSO) with cross-validation. Model performance was evaluated using the concordance index (C-index). Due to the nature of self-reported data and the difficulty participants had in distinguishing between OA and other forms of arthritis, OA was not included in the final multivariable model. In addition, OA lies on the causal pathway to JR and strongly dominates the outcome. Therefore, its exclusion allowed for a clearer interpretation of other determinants.

Missing covariate data were imputed using the mice package in R with predictive mean matching (pmm). A single imputation was performed (m = 1), and the imputation model included all covariates. Survival time and event indicator were fully observed and therefore not imputed. Covariates were imputed across all available waves (baseline and follow-up visits). Sensitivity analyses comparing imputed and complete-case models showed consistent results, with no substantive differences in effect estimates (Supplementary Table S1). Supplementary Table S2 reports the proportion of missingness per variable at each wave. Sex was used as a stratification variable in the model.

A secondary analysis was performed to explore the potential interaction between sex and SES. Investigating this interaction allowed for a more nuanced understanding of risk patterns and revealed possible sex-specific differences, given prior evidence that SES may influence health outcomes and JR differently between sexes [19]. In this model, sex and SES were used as covariates along with the interaction term. The interaction effect was further explored and visualised using marginal effect plots generated by the “emmeans” package in R.

A Fine–Gray subdistribution hazards model was fitted as a sensitivity analysis using baseline covariates only, treating death as a competing event. Fine–Gray models do not accommodate our time-dependent covariate structure and were therefore not used as the primary analysis. Because our primary aim was etiologic and required time-dependent covariates, we relied on time-dependent Cox models for the main analysis and interpreted the Fine–Gray results as complementary. We also conducted another sensitivity analysis to examine the robustness of our findings. Specifically, to address potential reverse causality related to participants with early symptoms of osteoporosis or OA, we re-estimated the models with adjustment for time-varying calcium supplement intake. This allowed us to assess whether the observed association between dietary calcium intake and JR risk persisted after accounting for supplement use. All statistical analyses were conducted using R Version 4.3.1 (R Foundation for Statistical Computing, Vienna, Austria).

## Results

In women, 135 of 1,446 (9.3%) had incident JR. Hip replacement was the prevailing procedure, accounting for 79 surgeries, followed by knee replacement with 56 surgeries. Similarly, 88 out of 1,436 men (6.1%) had undergone JR. Hip replacement was also the more common procedure among men, with 52 surgeries, and knee replacement with 36 surgeries.

Data for men and women were combined for analyses (*n* = 2,882), of which 223 (7.7%) JRs were identified. The median follow-up time was 16.7 years (IQR: 9.7–23.2). Table 1 compares baseline characteristics between participants with and without JR. Significant differences were observed in age, sex, BMI, waist circumference, spine T-score, osteoarthritis, previous fracture, and cardio-metabolic conditions. Participants with JR were generally older, with a higher proportion of JR among women (60.5%) compared to men (39.5%). The distribution of SES differed between participants. Among JR participants, 35.0% were in the low SES group, 22.9% in the medium SES group, and 42.2% in the high SES group. These results indicate that JR occurred disproportionately among individuals in the medium and high SES groups. Participants with JR had approximately 1.7 kg/m^2^ higher BMI and 3 cm higher waist circumference. Spine T-scores were significantly higher among those who had JR. Similarly, participants with JR were more likely to report a previous fracture and cardiometabolic conditions compared with those without JR. However, no significant differences were observed in smoking, alcohol intake, physical activity, dietary calcium intake, P1NP, CTX, pulmonary conditions, falls, and muscle weakness between the groups.

**Table 1:** Baseline characteristics of the participants included in the study (*n* = 2,882), stratified by joint replacement (JR) status

Variables	All (*n* = 2,882)	No JR (*n* = 2,659)	JR (*n* = 223)	*p*-value
**Demographic variables**				
Age (yr)	54.3 (37.8, 71.6)	53.4 (37.0, 71.5)	60.0 (49.2, 71.9)	< 0.001
Sex				0.001
- Men	1,436 (49.8%)	1,348 (50.7%)	88 (39.5%)	
- Women	1,446 (50.2%)	1,311 (49.3%)	135 (60.5%)	
SES				0.256
- Low	1,139 (39.5%)	1,061 (39.9%)	78 (35.0%)	
- Medium	563 (19.5%)	512 (19.3%)	51 (22.9%)	
- High	1,180 (40.9%)	1,086 (40.8%)	94 (42.2%)	
**Lifestyle factors**				
Smoking				0.138
- Nonsmoker	1,462 (50.7%)	1,351 (50.8%)	111 (49.8%)	
- Past smoker	960 (33.3%)	875 (32.9%)	85 (38.1%)	
- Current Smoker	460 (16.0%)	433 (16.3%)	27 (12.1%)	
Alcohol intake				0.921
- Low	1,973 (68.5%)	1,821 (68.5%)	152 (68.2%)	
- High	909 (31.5%)	838 (31.5%)	71 (31.8%)	
Physical activity				0.371
- Active	2,065 (71.7%)	1,911 (71.9%)	154 (69.1%)	
- Inactive	817 (28.3%)	748 (28.1%)	69 (30.9%)	
Dietary calcium intake (g/day)	0.8 (0.5, 1.0)	0.8 (0.5, 1.0)	0.7 (0.5, 1.0)	0.717
**Anthropometric/clinical measures**				
Height (cm)	167.6 (10.1)	167.7 (10.1)	166.0 (9.5)	0.014
Weight (kg)	75.3 (16.1)	75.1 (16.1)	78.3 (16.4)	0.004
BMI (kg/m^2^)	26.7 (4.9)	26.6 (4.8)	28.3 (5.2)	< 0.001
ALM/h^2^ (kg/m^2^)	7.5 (6.5, 8.6)	7.5 (6.5, 8.6)	7.4 (6.7, 8.7)	0.568
Waist circumference (cm)	91.0 (13.7)	90.7 (13.7)	93.7 (13.1)	0.002
Hip T-score	− 0.9 (1.2)	− 0.9 (1.2)	− 0.8 (1.2)	0.445
Spine T-score	− 0.3 (1.5)	− 0.3 (1.4)	− 0.1 (1.6)	0.017
Systolic BP (mm/Hg)	129.7 (20.5)	129.4 (20.5)	133.7 (20.3)	0.002
Diastolic BP (mm/Hg)	80.2 (13.0)	80.1 (13.0)	81.3 (13.0)	0.195
**Lipids (cholesterol)**				
Total Cholesterol (mmol/L)	5.3 (1.1)	5.3 (1.1)	5.4 (1.1)	0.121
LDL (mmol/L)	3.0 (0.9)	3.0 (0.9)	3.1 (0.9)	0.335
HDL (mmol/L)	1.3 (0.3)	1.3 (0.3)	1.3 (0.4)	0.691
Triglycerides (mmol/L)	1.3 (0.9, 1.8)	1.3 (0.9, 1.8)	1.3 (0.9, 1.9)	0.742
**Inflammatory marker**				
hsCRP (mg/L)	1.9 (0.9, 3.8)	1.9 (0.9, 3.8)	2.2 (1.0, 4.2)	0.822
**Bone turnover marker**				
P1NP (µg/L)	37.0 (27.0, 51.0)	37.0 (27.0, 51.0)	36.0 (26.0, 48.0)	0.943
CTx (ng/L)	334.9 (228.0, 474.8)	335.8 (228.7, 479.1)	327.0 (221.3, 450.2)	0.236
**Plasma glucose**				
Plasma glucose (mmol/L)	5.4 (1.1)	5.4 (1.1)	5.5 (0.9)	0.160
**Comorbid conditions**				
Osteoarthritis	275 (9.5%)	238 (9.0%)	37 (16.6%)	< 0.001
Fracture > 20 yr	1,241 (43.1%)	1,128 (42.4%)	113 (50.7%)	0.017
Falls in the past	634 (22.0%)	583 (21.9%)	51 (22.9%)	0.744
Diabetes	180 (6.2%)	163 (6.1%)	17 (7.6%)	0.376
Cardio-metabolic conditions	2,167 (75.2%)	1,979 (74.4%)	188 (84.3%)	0.001
Pulmonary conditions	465 (16.1%)	429 (16.1%)	36 (16.1%)	0.997
Cancer	306 (10.6%)	276 (10.4%)	30 (13.5%)	0.152
Muscle weakness	38 (1.3%)	35 (1.3%)	3 (1.3%)	0.971

Note: Values are presented as mean (standard deviation) for normally distributed continuous variables, median (IQR) for non-normally distributed continuous variables, and number (percentage) for categorical variables. P-values are based on: Independent t-tests for comparisons of means (continuous variables with normal distribution), Mann–Whitney U tests for non-normally distributed continuous variables (as applicable), and Chi-square tests (χ2) for categorical variables. Bold *p*-values indicate statistical significance at *p* < 0.05

*Abbreviation:* JR = Joint replacement; SES = Socioeconomic status (based on IRSD = Index of relative socioeconomic disadvantage); BMI = Body mass index; ALM/h^2^ = Appendicular lean mass adjusted for height squared; BMD = Bone mineral density at specific sites (s8 = spine, f0 = femoral neck), P1NP = procollagen type 1 N-terminal propeptide; hsCRP = high-sensitivity C-reactive protein; CTx = C-terminal telopeptide of type I collagen; HDL = high-density lipoprotein cholesterol, LDL = low-density lipoprotein cholesterol

### Multivariable model

Lower dietary calcium intake, lower spine BMD, lower P1NP, lower BMI, non-fallers, and no history of cancer were associated with reduced risk of JR (Table 2). Medium SES was linked to increased risk compared with lower SES. BMI and ALM/h^2^ were highly correlated and could not be included simultaneously; when BMI was substituted with ALM/h^2^, the ALM/h^2^ became statistically significant, suggesting a positive association with JR. When both were included, only one remained significant, likely reflecting collinearity. In exploratory analyses, stepwise Cox regression identified the same predictors as the multivariable model (C-index 0.64) (Table 3). Similarly, the LASSO model retained SES, physical activity, BMI, P1NP, falls, spine BMD, cancer, and muscle weakness. Despite modest discrimination (C-index 0.64), predictor selection was consistent across approaches, supporting the stability of the identified covariates.

**Table 2:** Time-dependent Cox proportional hazard regression model (multivariate), using sex stratification

Predictors	Categories	HR (BMI AM)	95%CI (BMI AM)	*p*-values (BMI AM)	HR (ALM/h^2^ AM)	95%CI (ALM/h^2^ AM)	*p*-values (ALM/h^2^ AM)
SES	Medium	1.52	1.07–2.16	**0.019**	1.51	1.07–2.15	**0.021**
	High	1.19	0.87–1.63	0.270	1.17	0.85–1.59	0.334
Smoking	Past	1.20	0.89–1.62	0.235	1.20	0.89–1.62	0.225
	Current	1.14	0.70–1.87	0.602	1.09	0.66–1.78	0.739
Alcohol intake	Low	1.05	0.77–1.42	0.769	1.06	0.78–1.44	0.706
Physical active	Active	0.84	0.62–1.14	0.264	0.80	0.59–1.07	0.138
Low dietary calcium intake (g/day)	**-**	0.74	0.52–1.04	0.085*	0.75	0.53–1.06	0.107
Low BMI (kg/m^2^)	**-**	0.96	0.94–0.99	**0.009**	**-**	**-**	**-**
Low ALM/h^2^ (kg/m^2^)	**-**	-	-	**-**	0.84	0.73–0.95	**0.008**
Low spine T-score	**-**	0.84	0.77–0.92	** < 0.001**	0.83	0.76–0.91	** < 0.001**
Low P1NP (µg/L)	**-**	0.69	0.50–0.96	**0.026**	0.70	0.51–0.97	**0.033**
hsCRP (mg/L)	**-**	0.99	0.96–1.01	0.328	0.99	0.97–1.02	0.549
Low plasma glucose	-	1.00	0.58–1.74	0.995	0.97	0.56–1.68	0.922
Non-fallers	-	0.74	0.55–0.99	**0.043**	0.73	0.55–0.98	**0.036**
No prior fracture	-	1.05	0.75–1.46	0.797	1.06	0.75–1.48	0.751
No cardio-metabolic disease	-	0.99	0.66–1.47	0.952	0.95	0.64–1.42	0.808
No cancer	-	0.66	0.48–0.90	**0.008**	0.65	0.48–0.89	**0.007**
No pulmonary conditions	-	0.97	0.69–1.36	0.857	0.95	0.68–1.363	0.766
No muscle weakness	-	1.86	0.45–7.57	0.389	1.81	0.44–7.39	0.407

P-values are derived from a time-dependent Cox proportional hazards regression model. *HR* > *1 indicates increased risk; HR* < *1 indicates reduced risk. Comorbidities are referenced against the absence or the lowest-risk group. Hazard ratios for continuous predictors represent the relative risk of joint replacement per 1-unit decrease (BMI and ALM/h*^*2*^* per 1 kg/m*^*2*^* decrease; P1NP per 1 µg/L decrease; dietary intake per 1 g/day decrease). Reverse-coding was applied to highlight protective associations. Variables were analysed in their original units and were not standardised*. *P-values* < *0.05 are considered statistically significant. Model 1 adjusted for BMI; Model 2 adjusted for ALM/h*^*2*^. *P-values between 0.05 and 0.10 are interpreted as marginally significant and marked with an asterisk (*)

*Abbreviations:* Hazard ratios (HRs); 95% confidence intervals (CIs); SES = Socioeconomic Status (based on IRSD: Index of relative socioeconomic disadvantage); BMI = Body mass index; ALM/h^2^ = Appendicular lean mass adjusted for height in meter square; BMD = Bone mineral density (measured at the spine); P1NP = Procollagen type 1 N-terminal propeptide; hsCRP = high-sensitivity C-reactive protein; AM = Adjusted Model

**Table 3:** Time-dependent Cox regression model with selected variables using stepwise forward selection

Predictors	Categories	HR	95%CI	*p*-values
SES	Medium	1.51	1.07–2.14	**0.020**
	High	1.17	0.86–1.60	0.310
Low dietary calcium intake (g/day)	**-**	0.74	0.53–1.05	0.093*
Low BMI (kg/m^2^)	**-**	0.96	0.94–0.99	**0.004**
Low spine T-score	**-**	0.84	0.77–0.92	** < 0.001**
Low P1NP (µg/L)	-	0.69	0.50–0.95	**0.024**
Non-fallers	-	0.74	0.56–0.99	**0.046**
No cancer	-	0.64	0.47–0.88	**0.005**

P-values are derived from a time-dependent Cox proportional hazards regression model. *HR* > *1 indicates increased risk; HR* < *1 indicates reduced risk. Comorbidities are referenced against the absence or the lowest-risk group. Hazard ratios for continuous predictors represent the relative risk of joint replacement per 1-unit decrease (BMI and ALM/h*^*2*^* per 1 kg/m*^*2*^* decrease; P1NP per 1 µg/L decrease; dietary intake per 1 g/day decrease). Reverse-coding was applied to highlight protective associations. Variables were analysed in their original units and were not standardised. P-values* < *0.05 are considered statistically significant*

^*^P-values between 0.05 and 0.10 are interpreted as marginally significant and marked with an asterisk (*)

*Abbreviations:* Hazard ratios (HRs); 95% confidence intervals (CIs); SES = Socioeconomic Status (based on IRSD = Index of relative socioeconomic disadvantage); BMI = Body mass index; BMD = Bone Mineral Density (measured at the spine); P1NP = Procollagen type 1 N-terminal propeptide

When stratified by joint site, several predictors showed differential associations. BMI was strongly associated with knee replacement (HR 0.94, 95% CI, 0.91–0.96, *p* < 0.001), but not with hip replacement (HR 1.01, 95% CI 0.99–1.04, *p* < 0.345). In contrast, P1NP showed a stronger association with hip replacement (HR 0.55, 95% CI 0.29–1.03, *p* = 0.063) than with knee replacement (HR 0.76, 95% CI 0.31–1.87, *p* = 0.555). Falls and prior fracture history were significant predictors of hip replacement but not knee replacement. These findings suggest mechanical drivers such as BMI are more relevant for knee OA, whereas biological and fragility-related factors (P1NP, fractures, falls) appear more important for hip OA (Table 4). In age-stratified analyses, BMI showed different patterns. Among participants aged 40–59 years, lower BMI was associated with reduced risk of JR (HR 0.97, 95% CI, 0.93–1.01, *p* = 0.115). Among those aged ≥ 60 years, lower BMI was associated with increased risk (HR 1.04, 95% CI 1.00–1.08, *p* = 0.075). Although both associations were borderline significant, the direction of effect suggests BMI reduction in midlife may confer protection, whereas in older adults, low BMI may be linked to a higher risk (Table 5).

**Table 4:** Time-dependent Cox proportional hazards regression (multivariable), stratified by joint site (knee and hip)

Predictors	Categories	HR (Hip)	95%CI (Hip)	*p*-values (Hip)	HR (Knee)	95%CI (Knee)	*p*-values (Knee)
SES	Medium	1.51	1.09–2.09	**0.014**	1.22	0.79–1.87	0.369
	High	1.05	0.78–1.42	0.742	1.45	1.03–2.05	**0.035**
Smoking	Past	1.18	0.88–1.58	0.266	1.21	0.87–1.70	0.259
	Current	1.27	0.84–1.90	0.256	1.02	0.60–1.73	0.933
Alcohol intake	Low	1.21	0.90–1.63	0.216	1.13	0.79–1.62	0.492
Physical active	Active	1.06	0.78–1.44	0.700	0.91	0.64–1.28	0.571
Low dietary calcium intake (g/day)	**-**	0.73	0.54–1.00	**0.048**	0.79	0.53–1.17	0.235
Low BMI (kg/m^2^)	**-**	1.01	0.99–1.04	0.345	0.94	0.91–0.96	** < 0.001**
Low spine T-score	**-**	0.85	0.77–0.92	** < 0.001**	0.86	0.77–0.96	**0.005**
Low P1NP (µg/L)	**-**	0.55	0.29–1.03	0.063	0.76	0.31–1.87	0.555
hsCRP (mg/L)	**-**	1.00	0.98–1.02	0.970	0.99	0.97–1.02	0.625
Low plasma glucose	-	1.09	0.59–2.01	0.781	1.23	0.64–2.37	0.540
Non-fallers	-	0.80	0.60–1.06	0.124	0.83	0.59–1.17	0.283
No prior fracture	-	0.62	0.47–0.82	**0.001**	0.84	0.58–1.21	0.350
No cardio-metabolic disease	-	0.94	0.65–1.34	0.715	0.60	0.36–1.00	0.051
No cancer	-	0.90	0.64–1.26	0.540	0.69	0.47–1.01	0.056
No pulmonary conditions	-	1.09	0.77–1.54	0.622	0.96	0.65–1.39	0.814
No muscle weakness	-	0.87	0.32–2.37	0.785	2.51	0.35–18.08	0.362

P-values are derived from a time-dependent Cox proportional hazards regression model. *HR* > *1 indicates increased risk; HR* < *1 indicates reduced risk. Comorbidities are referenced against the absence or the lowest-risk group. Hazard ratios for continuous predictors represent the relative risk of joint replacement per 1-unit decrease (BMI and ALM/h*^*2*^* per 1 kg/m*^*2*^* decrease; P1NP per 1 µg/L decrease; dietary intake per 1 g/day decrease). Reverse-coding was applied to highlight protective associations. Variables were analysed in their original units and were not standardised*. *P-values* < *0.05 are considered statistically significant*

^*^P-values between 0.05 and 0.10 are interpreted as marginally significant and marked with an asterisk (*). *Model 1 for hip replacements; Model 2 for knee replacements*

*Abbreviations:* Hazard ratios (HRs); 95% confidence intervals (CIs); SES = Socioeconomic Status (based on IRSD: Index of relative socioeconomic disadvantage); BMI = Body mass index; ALM/h^2^ = Appendicular lean mass adjusted for height in meter square; BMD = Bone mineral density (measured at the spine); P1NP = Procollagen type 1 N-terminal propeptide; hsCRP = high-sensitivity C-reactive protein

**Table 5:** Time-dependent Cox proportional hazards regression (multivariable), stratified by age group

Predictors	Categories	HR (40–59 yrs)	95%CI (40–59 yrs)	*p-*values (40–59 yrs)	HR (60 + yrs)	95%CI (60 + yrs)	*p*-values (60 + yrs)
SES	Medium	2.07	1.23–3.48	**0.006**	1.16	0.74–1.84	0.513
	High	0.91	0.54–1.53	0.724	1.16	0.79–1.70	0.456
Smoking	Past	1.09	0.68–1.75	0.727	1.18	0.80–1.73	0.410
	Current	0.90	0.48–1.66	0.725	2.04	1.15–3.61	**0.015**
Alcohol intake	Low	0.90	0.56–1.46	0.682	1.37	0.93–2.04	0.114
Physical active	Active	1.23	0.70–2.14	0.473	1.01	0.69–1.46	0.979
Low dietary calcium intake (g/day)	**-**	0.76	0.47–1.22	0.257	0.63	0.41–0.98	**0.039**
Low BMI (kg/m^2^)	**-**	0.97	0.93–1.01	0.115	1.04	1.00–1.08	0.075
Low spine T-score	**-**	0.92	0.78–1.08	0.304	0.83	0.74–0.92	**0.001**
Low P1NP (µg/L)	**-**	1.26	0.17–9.15	0.822	0.43	0.22–0.86	**0.017**
hsCRP (mg/L)	**-**	0.97	0.91–1.03	0.277	1.00	0.98–1.03	0.741
Low plasma glucose	-	-	-	**-**	0.77	0.41–1.43	0.405
Non-fallers	-	0.97	0.57–1.64	0.909	0.73	0.51–1.05	0.091
No prior fracture	-	0.71	0.43–1.17	0.180	0.56	0.39–0.80	**0.002**
No cardio-metabolic disease	-	1.08	0.66–1.78	0.753	0.82	0.46–1.46	0.502
No cancer	-	1.63	0.65–4.06	0.298	0.75	0.52–1.10	0.139
No pulmonary conditions	-	0.83	0.49–1.42	0.499	1.31	0.81–2.12	0.262
No muscle weakness	-	0.30	0.09–1.00	**0.049**	2.48	0.34–18.12	0.370

P-values are derived from a time-dependent Cox proportional hazards regression model. *HR* > *1 indicates increased risk; HR* < *1 indicates reduced risk. Comorbidities are referenced against the absence or the lowest-risk group. Hazard ratios for continuous predictors represent the relative risk of joint replacement per 1-unit decrease (BMI and ALM/h*^*2*^* per 1 kg/m*^*2*^* decrease; P1NP per 1 µg/L decrease; dietary intake per 1 g/day decrease). Reverse-coding was applied to highlight protective associations. Variables were analysed in their original units and were not standardised*. *P-values* < *0.05 are considered statistically significant*

^*^P-values between 0.05 and 0.10 are interpreted as marginally significant and marked with an asterisk (*). *Model 1 for participants 40–59 years; Model 2 for participants 60* + *years*

*Abbreviations:* Hazard ratios (HRs); 95% confidence intervals (CIs); SES = Socioeconomic Status (based on IRSD: Index of relative socioeconomic disadvantage); BMI = Body mass index; ALM/h^2^ = Appendicular lean mass adjusted for height in meter square; BMD = Bone mineral density (measured at the spine); P1NP = Procollagen type 1 N-terminal propeptide; hsCRP = high-sensitivity C-reactive protein

**Fig. 2:**
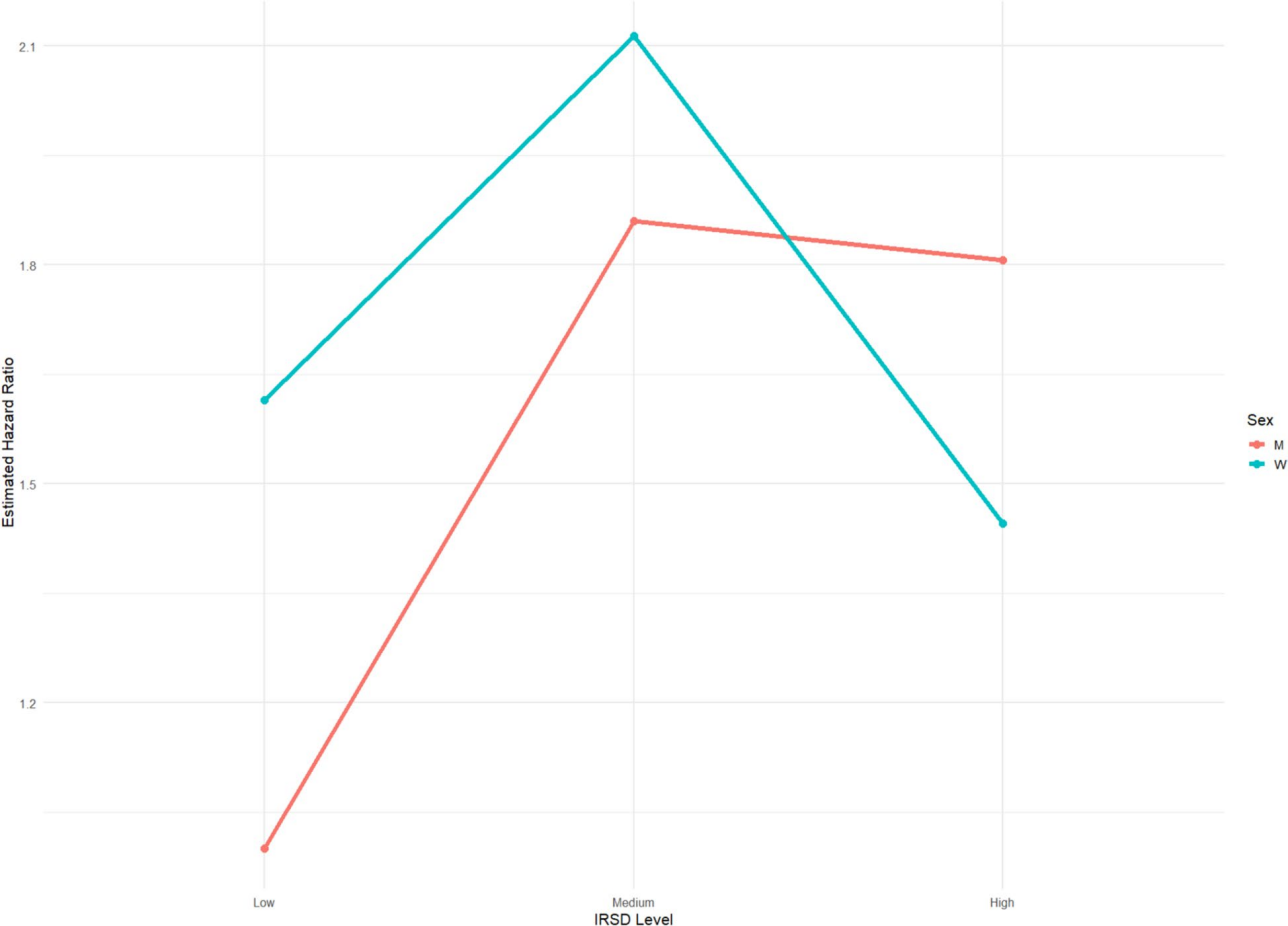
Marginal means plot showing the interaction between socioeconomic status (IRSD level) and sex on the hazard of joint replacement. *Hazard ratios are derived from a secondary time-dependent Cox proportional hazards model containing a sex-by-SES interaction term. This figure is presented to explore the effect modification

To examine the relationship between SES and JR in relation to sex, a separate model was fitted to test effect modification. Medium and high SES were associated with increased JR risk (HR 1.91, 95% CI, 1.04–3.50, *p* = 0.031; HR 1.88, 95% CI, 1.13–3.15, *p* = 0.013). A significant interaction between sex and high SES (HR 0.48, 95% CI, 0.25–0.91, *p* = 0.028) indicated that the association was stronger among men (Fig. 2). To complement these relative measures, Table 6 presents absolute incidence rates of JR per 1,000 person-years, stratified by SES and sex. Men with low SES served as the reference group. Absolute rates ranged from 2.76 in men with low SES to 6.02 in women with medium SES. Compared to men with low SES, women with low SES had more than double the risk (HR 2.29, 95% CI, 1.38–3.79, *p* = 0.001), while women with high SES had significantly lower risk (HR 0.42, 95% CI, 0.22–0.80, *p* = 0.008). Men with medium SES also showed elevated risk (HR 1.96, 95% CI, 1.07–3.58, *p* = 0.028). These findings highlight that both SES and sex contribute to differences in JR incidence and risk.

**Table 6:** Absolute incidence rates and hazard ratios for joint replacement risk by socioeconomic status and sex

SES	Sex	Events	Person-years	Incidence rate (per 1,000 PY)	HR	95%CI	*p-*values
Low	M	22.00	7,967.62	2.76	Reference	Reference	Reference
Low	W	51.00	12,069.46	4.23	2.29	1.38–3.79	0.001
Medium	M	20.00	4,208.50	4.75	1.96	1.07–3.58	0.028
Medium	W	38.00	6,309.89	6.02	0.69	0.34–1.41	0.313
High	M	46.00	9,851.97	4.67	1.82	1.09–3.05	0.023
High	W	46.00	11,868.80	3.88	0.42	0.22–0.80	0.008

Incidence rates are expressed per 1,000 person-years. Hazard ratios (HRs) and 95% confidence intervals (CIs) were estimated using time-dependent Cox proportional hazards regression models. The reference category is low socioeconomic status (SES) men. Models were adjusted for age and other covariates as specified in the statistical analysis section

*Abbreviations: M* = *Men; W* = *Women; SES* = *Socioeconomic Status; HR* = *Hazard Ratio; CI* = *Confidence Interval; PY* = *Person-years*

In the fully adjusted Fine-Gray competing risk model, participants with a history of cancer did not have a significantly different subdistribution hazard of JR compared to those without cancer (HR 0.92, 95% CI, 0.61–1.39, *p* = 0.70). Results for other covariates were directionally consistent with the time-dependent Cox models. To address potential reverse causality related to supplement use, an additional sensitivity analysis showed the association between dietary calcium intake and JR risk was attenuated and no longer significant after adjustment for calcium supplement intake (HR 0.75, 95% CI, 0.53–1.06, *p* = 0.108), suggesting supplement use may partly explain the marginal association observed previously (Table 7). When OA was included in the multivariable models, it was strongly associated with increased JR risk (HR 1.89, 95% CI 1.37–2.49, *p* = 0.001). Although this adjustment did not materially alter the overall findings, it attenuated the strength of the association with P1NP, which shifted from statistical significance to borderline (HR 0.55, 95% CI 0.30–1.02, *p* = 0.060). The direction of effect for P1NP remained consistent across all models.

**Table 7:** Time-dependent Cox proportional hazards regression (multivariable), adjusted for calcium supplementation

Predictors	Categories	HR	95%CI	*p*-values
SES	Medium	1.58	1.12–2.24	**0.010**
	High	1.22	0.89–1.67	0.210
Smoking	Past	1.19	0.88–1.61	0.251
	Current	1.13	0.69–1.85	0.628
Alcohol intake	Low	1.06	0.78–1.45	0.701
Physical active	Active	0.86	0.63–1.15	0.307
Low dietary calcium intake (g/day)	**-**	0.75	0.53–1.06	0.108
Low BMI (kg/m^2^)	**-**	0.97	0.94–1.00	**0.030**
Low spine T-score	**-**	0.83	0.76–0.91	** < 0.001**
Low P1NP (µg/L)	**-**	0.49	0.27–0.91	**0.024**
hsCRP (mg/L)	**-**	1.00	0.98–1.02	0.903
Low plasma glucose	-	0.87	0.52–1.46	0.594
Non-fallers	-	0.72	0.54–0.97	**0.029**
No prior fracture	-	1.05	0.75–1.47	0.764
No cardio-metabolic disease	-	0.97	0.65–1.45	0.894
No cancer	-	0.65	0.48–0.89	**0.006**
No pulmonary conditions	-	0.99	0.71–1.40	0.974
No muscle weakness	-	1.75	0.43–7.14	0.436
Calcium supplement use	-	1.25	0.84–1.84	0.266

P-values are derived from a time-dependent Cox proportional hazards regression model. *HR* > *1 indicates increased risk; HR* < *1 indicates reduced risk. Comorbidities are referenced against the absence or the lowest-risk group. Hazard ratios for continuous predictors represent the relative risk of joint replacement per 1-unit decrease (BMI and ALM/h*^*2*^* per 1 kg/m*^*2*^* decrease; P1NP per 1 µg/L decrease; dietary intake per 1 g/day decrease). Reverse-coding was applied to highlight protective associations. Variables were analysed in their original units and were not standardised*. *P-values* < *0.05 are considered statistically significant*

^*^P-values between 0.05 and 0.10 are interpreted as marginally significant and marked with an asterisk (*)

*Abbreviations:* Hazard ratios (HRs); 95% confidence intervals (CIs); SES = Socioeconomic Status (based on IRSD: Index of relative socioeconomic disadvantage); BMI = Body mass index; ALM/h^2^ = Appendicular lean mass adjusted for height in meter square; BMD = Bone mineral density (measured at the spine); P1NP = Procollagen type 1 N-terminal propeptide; hsCRP = high-sensitivity C-reactive protein

## Discussion

The findings of this study present a multifactorial approach to identify the key predictors of JR in weight-bearing joints. The results indicate that lower dietary calcium intake, lower spine BMD, lower BMI, lower P1NP levels, absence of falls, and no history of cancer were each associated with a reduced risk of JR. Participants in the low SES group had a lower risk of JR than those in the medium and high SES groups.

As reported in the previous literature, age was a risk factor for JR, with younger adults being more protected than older adults [20]. Given the age-dependent nature of JR, age was used as the primary time scale rather than time since study entry. This approach enabled comparisons between individuals of the same age, accounted for differences in age at entry, and better reflected lifetime exposure to relevant covariates. Studies have shown that men are less likely to have JR compared to women [19, 21]. In our study, rather than adjusting for sex as a covariate, the model was adjusted for sex by stratification. Thus, the model accounted for sex-specific risks while providing unbiased effect-size estimates for other covariates and avoided imposing the assumption that sex-specific estimates were constant over time.

Regarding SES, our results showed that participants in the medium and high SES groups had a higher risk of JR than those in the low SES group. The effect estimate for higher SES was more pronounced among men and nearly halved among women. Marginal means plot confirmed that the interaction was inconsistent across SES groups. By presenting both hazard ratios and absolute incidence rates, we provide a clearer picture of the SES × sex interaction. Hazard ratios quantify relative risk after adjustment, while absolute rates highlight the clinical burden in each subgroup. For example, medium SES women had the highest absolute incidence, yet their relative risk compared with low SES men was not significantly elevated. Conversely, high SES women had lower absolute rates and a significantly reduced relative risk. This dual presentation underscores that SES and sex jointly shape the burden of JR, and that absolute rates are essential for clinical interpretation beyond relative measures.

Although it might be expected that individuals from higher SES backgrounds would have better health and therefore a lower risk of JR [22]. The opposite was observed. This pattern may be explained by inequitable access to healthcare, with financial capacity and private insurance driving surgical uptake rather than disease burden [23]. As reported in the 2025 Australian Orthopaedic Association National Joint Replacement Registry annual report, 60–70% of JRs were performed in private hospitals, disproportionately among higher-SES individuals [24]. This highlights that the association between SES and JR reflects affordability and access to private surgery, rather than SES as a biological risk factor. Importantly, this also implies a significant unmet surgical need in disadvantaged populations, where disease prevalence may be similar but access to surgery is limited. Policy-level investigations into healthcare equity and underservicing among low SES groups are therefore warranted. While we observed that men in high SES groups had a greater likelihood of surgery compared with women, the mechanisms underlying this sex-specific difference remain unclear. Prior literature does not provide a definitive explanation. We therefore interpret this cautiously, noting that gender differences in health literacy [25], attitudes toward surgery and recovery [26, 27], different activity patterns, and delayed treatment behaviour may contribute to the higher risk of JR observed among men in higher SES groups [27]. Further research is needed to substantiate these hypotheses.

When anthropometric measures were assessed, our results revealed that the risk of JR reduced by 4% for each 1 kg/m^2^ decrease in BMI. This finding is consistent with previous studies, which showed that higher BMI was strongly associated with joint degeneration and OA, particularly in weight-bearing joints [28, 29]. The implausible association observed between lower ALM/h^2^ and reduced JR risk might suggest that ALM could be acting as a surrogate marker for overall body weight. A link between lower ALM and lower risk of clinically diagnosed and symptomatic knee OA among older men was previously reported in a prospective cohort study conducted in the USA. There was no evidence of a significant relationship in older women [30]. While JR is a clinically relevant endpoint, it also reflects healthcare utilisation. Therefore, the associations observed must be viewed in the context of healthcare utilisation as well as biological pathways.

Turning to lifestyle factors, it was previously reported that lifestyle determinants play a role in reducing the risk of JR [31]. No association was detected between smoking, alcohol consumption, or physical activity and JR in our cohort. Lower dietary calcium intake was associated with a reduced risk of JR. This association may reflect confounding by indication, as individuals with early symptoms of osteoporosis or OA are often advised to increase calcium intake, raising the possibility of reverse causality [32–34]. While calcium is essential for bone health, excessive intake or disruption of calcium homeostasis has been linked to accelerated joint degeneration [35]. Importantly, the marginal association observed in the main model did not persist after adjustment for supplement use in a sensitivity analysis. This attenuation supports the likelihood of reverse causality, whereby those with existing musculoskeletal concerns are more likely to consume calcium supplements or high-calcium diets. Our findings suggest that calcium intake was not independently associated with reduced risk after accounting for supplement use, underscoring the importance of accounting for supplement behaviours in nutritional epidemiology. Taken together, these results indicate that calcium intake alone may not confer reduced risk against musculoskeletal outcomes, and observed associations may be driven by reverse causality or confounding.

Another important finding showed that lower spine BMD can be a predictor of a healthy joint state, which appears counterintuitive [36]. Mostly, higher BMD is considered a healthy state, but in this situation, it might reveal degenerative changes in the spine, thereby indicating a more advanced stage of OA [37]. One finding from a prospective cohort study showed that higher spine BMD was associated with an increased risk of progression of medial, lateral, and tibiofemoral cartilage defects [38]. However, interpretation of factors associated with a reduced risk of JR requires caution, as these variables may function as markers of underlying disease processes rather than true causal mechanisms of risk reduction. For example, higher spine BMD may reflect degenerative changes such as osteophytes, subchondral sclerosis, or other degenerative changes rather than true skeletal health. All of which are characteristic of established OA, which artificially elevate DXA-derived BMD [39–41]. Therefore, the observed association may be partly explained by age-related increases in spine BMD, particularly among men, as previously reported in the Geelong Osteoporosis Study [42]. Consequently, individuals with lower BMD may simply have fewer degenerative structural changes at baseline, indicating a correlate of underlying clinical states rather than an independent risk-reducing factor. It is worth noting that JR represents both a clinical endpoint and a healthcare-utilisation outcome. Therefore, the observed association highlights the role of healthcare access and patient preference, in addition to biological disease progression. Accordingly, the interpretation remains tentative given the outcome definition.

Additionally, given the importance of bone turnover markers in musculoskeletal health, our findings revealed that lower levels of P1NP were associated with a reduced risk of JR. Although P1NP is primarily a marker of bone formation, elevated concentrations suggest increased bone turnover, thereby indicating increased bone loss [43]. Previous studies have associated P1NP with adverse health outcomes, but few have linked P1NP directly to knee or hip replacement [43, 44]. Importantly, elevated P1NP may also reflect active subchondral bone remodelling in early-to-mid-stage OA, a process characterised by increased turnover and sclerosis that precede cartilage degeneration [45–48]. Such remodelling has been implicated in OA progression and may contribute to the increased risk of JR. Therefore, our findings support the possibility that altered bone metabolism, particularly subchondral bone changes, may explain the observed associations with JR and highlight the potential utility of P1NP as a biomarker for identifying individuals at risk of progression. Although after adjusting for OA, the observed association between P1NP and JR risk was attenuated to borderline significance, likely reflecting shared variance, as OA progression involves bone remodelling processes captured by P1NP. Future studies should explore models both with and without OA adjustment to disentangle biomarker effects from OA-related pathways. However, the association between P1NP and JR risk may reflect not only a surrogate marker of OA severity, but also factors such as healthcare access, patient preferences, and clinical decision-making.

Comorbid conditions further contributed to variation in JR risk, independent of the above-mentioned determinants. Specifically, participants who were non-fallers and cancer-free had a lower risk for JR. The association between non-fallers and the reduced risk of JR might be influenced by the existing relationship between falls and the development of OA [49]. Previous research has shown that individuals with greater muscle mass, neuromuscular control, and overall physical fitness are less likely to experience falls, which are a known precursor to joint deterioration and subsequent JR [50]. Falls can also contribute to joint damage, thereby increasing the likelihood of JR. On the other hand, individuals with OA may be more likely to fall due to pain, stiffness, and impaired mobility, suggesting a bidirectional relationship between OA and falls [51]. Although OA is not the only source of JR in this study, it remains significantly associated with increased JR risk. Importantly, JR should be understood as a healthcare utilisation outcome, not simply a reflection of underlying OA severity. Moreover, evidence linking JR with cancer is limited. In our analysis, a significant association between no history of cancer and reduced risk of JR was observed, but this was attenuated in the Fine-Gray model, likely reflecting the higher competing risk of death among participants with cancer, which reduces their probability of undergoing elective JR, even though cancer patients could share inflammatory pathways with OA [52] or functional decline and frailty [53]. Still, the observed association requires careful consideration.

Taken together, stratified analyses by joint site revealed that mechanical drivers such as BMI were more strongly associated with knee replacement. In contrast, biological and fragility-related factors, such as P1NP, falls, and prior fracture, were more relevant to hip replacement. These findings underscore that hip and knee OA may progress through distinct pathways, with mechanical load predominating in the knee and systemic or bone-related factors appearing more influential in the hip [54]. This heterogeneity highlights the need to consider joint-specific mechanisms when evaluating risk factors and designing preventive strategies. By emphasising these differences, our study contributes to a more nuanced understanding of OA progression and its translation into clinical decision-making. In addition to stratification by joint site, the age-specific analyses provide context for potential risk factors. In mid-life (40–59 years), lower BMI was associated with reduced risk of JR, consistent with the role of mechanical load in OA progression [54, 55]. However, in older adults (≥ 60 years), lower BMI was associated with increased risk, which may reflect frailty, sarcopenia, or unintentional weight loss due to comorbidities [56, 57]. These subgroup findings are exploratory and should be interpreted cautiously, as the estimates were borderline, possibly due to limited sample size or low event rate. This divergence explains why BMI was significant in the pooled model but only borderline in stratified models: the overall effect is driven by protective associations in mid-life, while later life introduces competing biological mechanisms. These findings highlight that the timing of BMI reduction is critical; intervention earlier in adulthood may be more effective in reducing JR risk, whereas in older age, low BMI may signal vulnerability rather than protection.

### Strengths and limitations

An important strength of this study is the use of time-dependent covariates, in which the dynamic nature of each covariate was captured in the analysis. For example, changes in BMI, physical activity, or comorbid conditions were incorporated at each follow-up wave, rather than assuming these exposures remained constant throughout the study period. This approach helped improve model accuracy. Expressing age as the primary time scale also provides an accurate estimate of risk factors related to the ageing process, making the results more interpretable in a clinical context. Another strength is the use of a large cohort of men and women, with approximately 16 years of follow-up. Information about the participants was obtained before the outcome occurred. This presents a comprehensive assessment of JR determinants over an extended period. Model discrimination was modest, with C-indices of approximately 0.64 for the variable selection model and 0.66 for the multivariable model. These values are consistent with other epidemiological studies of musculoskeletal outcomes, in which predictive models for JR or related events typically achieve C indices in the range of 0.60–0.66 [58, 59]. This reflects the multifactorial nature of JR risk, where single models rarely achieve high predictive accuracy. Importantly, the consistency of predictors across stepwise and penalised regression approaches supports the robustness of the findings. These results contribute to understanding risk and protective factors for JR, which may inform clinicians and public health strategies to reduce the risk of JR. However, the model is intended to identify population-level associations and potential pathways rather than to serve as a predictive tool for clinical decision-making.

A limitation of this study is that the proportion of participants who had JR was relatively small, possibly resulting in a type II error. The outcomes (JR) could not be classified by indication (e.g., OA versus fracture) or laterality. While most procedures were attributable to OA, we cannot exclude other causes. This restricts our ability to interpret OA-specific determinants of JR, and future studies with more detailed outcome classification are warranted. Another limitation of this study is that occupational exposure was assessed at single time points and not updated over time. Prior injury and joint pain severity, which are established drivers of JR, were also unavailable. Although our models adjusted for multiple covariates, residual confounding from these unmeasured or time-varying factors may remain. Future studies incorporating repeated measures of occupation and joint pain severity will be important to disentangle their contribution alongside biomarkers and comorbidities. Stratification by age and joint site reduced sample size and statistical power, which may explain why BMI was significant in the pooled model but only borderline in subgroup analyses. BMI was used as a proxy for adiposity and does not distinguish between fat and lean mass; in older adults, low BMI may reflect frailty or sarcopenia rather than reduced mechanical load. Participants younger than 40 contributed person-time but no events, limiting precision in early-adulthood estimates. Furthermore, lifestyle predictors and some comorbidities were self-reported, which may also contribute to potential data quality issues. However, objective measures, including densitometry, anthropometry, data linkage, clinical measures, and biochemistry, were utilised where possible.

Additionally, the timing of falls relative to OA diagnosis could not be established. Falls were recorded as occurring within the past year, while OA was captured as a binary variable. Consequently, we cannot determine whether falls preceded or followed OA onset. This restricts our ability to fully interpret the causal direction of the observed association, and future studies with more detailed temporal data are warranted. We also acknowledge that single imputation (m = 1) for covariates is not an optimal approach. However, survival time and event indicator were fully observed, and not imputed, and complete-case sensitivity analyses produced consistent results, providing reassurance regarding the robustness of our findings. In the primary multivariable model, OA was excluded to avoid adjusting for a variable on the causal pathway. However, this choice may limit clinical interpretability, since OA is the principal indication for JR. In sensitivity analyses, including OA yielded consistent findings. This supports the robustness of the primary model, which identifies upstream determinants of JR risk. The sensitivity analysis complements this by situating those determinants within the clinical context of OA progression. The study was conducted in southeastern Australia. As such, the results may not translate to other geographical regions with different demographic characteristics and access to JR. Future research is needed to address these limitations and enhance the validity and generalisation of the findings. Additionally, further research is needed to elucidate the mechanisms by which higher P1NP levels influence joint health and the need for JR. Future studies should also explore the influence of SES and sex-related disparities among individuals undergoing JR in diverse populations. Recognising and understanding these dynamics is crucial because they allow clinicians, researchers, and policy makers to anticipate disease progression and intervene earlier. This, in turn, supports the development of targeted strategies that can slow joint degeneration and reduce the need for JR.

## Conclusion

A multifactorial approach was used to identify key determinants of JR using data from a large, randomly selected, age-stratified population-based cohort of Australian men and women. Our results indicate that lower body weight and lower P1NP levels were associated with a reduced risk of JR, suggesting several potential intervention pathways. Absence of falls and no history of cancer were associated with a reduced risk of JR in the multivariable model, although the association between cancer and JR was not statistically significant in the Fine-Gray sensitivity analysis, likely reflecting the higher competing risk of death among participants with cancer. Lower spine BMD and lower dietary calcium intake were associated with a reduced risk of JR; however, these findings should not be interpreted as biologically protective. Higher spine BMD often reflects osteophytes and subchondral sclerosis in established OA, which can artificially elevate DXA measurements and signal more advanced joint degeneration. Similarly, higher calcium intake may reflect clinical advice or treatment among individuals already identified as being at higher musculoskeletal risk, introducing confounding by indication and potential reverse causality. Overall, calcium intake was not independently associated with reduced risk after adjustment for supplement use, underscoring the need to consider supplement behaviours when interpreting dietary associations. These associations, therefore, likely represent markers of a healthier joint state at baseline, rather than true protective mechanisms. In addition, addressing sex differences and reducing barriers among individuals from different socioeconomic groups may support more equitable access to JR.

Our findings are consistent with the possibility that hip and knee OA may progress through distinct pathways, with mechanical load potentially predominating in the knee and systemic or bone-related factors playing a greater role in the hip. However, this interpretation is plausible but not definitive, given that JR could not be classified by indication. Moreover, age-specific analyses suggested differing associations between BMI and JR risk: lower BMI in mid-life was associated with reduced risk, whereas in older adults, lower BMI was associated with higher risk. The findings are exploratory and warrant further investigation before drawing practical implications. They also highlight the importance of considering joint site and age when interpreting risk factors and designing preventive strategies for JR.

In interpreting these findings, it is essential to recognize that JR is not solely a biological endpoint but also reflects healthcare access, patient preference, and clinical decision-making. This underscores the need for caution when linking observed associations to disease mechanisms alone.

## Supplementary Information

Supplementary Material 1.

## Data Availability

Access to the data is restricted and available only upon reasonable request.

## Abbreviations

ALM/h2: Appendicular lean mass adjusted for height squared
BMD: Bone mineral density
BMI: Body mass index
IRSD: Index of Relative Socioeconomic Disadvantage
SES: Socioeconomic status
LogReg: Logistic regression
PMM: Predictive mean matching
JR: Joint replacement
OA: Osteoarthritis
HR: Hazard ratio
CI: Confidence interval
AIC: Akaike Information Criterion
BIC: Bayesian Information Criterion
IQR: Interquartile range
C-index: Concordance index
LASSO: Least Absolute Shrinkage and Selection Operator
GOS: Geelong Osteoporosis Study
DXA: Dual-energy X-ray absorptiometry
AIHW: Australian Institute of Health and Welfare
