# Supplementary material for: Key determinants for lowering the risk of joint replacement in weight-bearing joints: a population-based cohort study
Source: Arthroplasty. 2026 Jun 8;8:45. doi: 10.1186/s42836-026-00403-9 (PMC13244897; doi:10.1186/s42836-026-00403-9)
Supplement: Supplementary file 1 — Supplementary Material 1. [file 42836_2026_403_MOESM1_ESM.docx]

**Supplementary material**

**Table S1**. Time-dependent Cox proportional hazards regression (multivariable), complete-case analysis before imputation.

| **Predictors** | **Categories** | **HR** | **95%CI** | ***p*-values** |
| --- | --- | --- | --- | --- |
| SES | Medium | 1.77 | 1.17–2.70 | **0.007** |
|  | High | 1.28 | 0.88–1.88 | 0.199 |
| Smoking | Past | 1.11 | 0.77–1.60 | 0.584 |
|  | Current | 0.90 | 0.49–1.66 | 0.742 |
| Alcohol intake | Low | 1.04 | 0.72–1.50 | 0.847 |
| Physical active | Active | 0.77 | 0.54–1.10 | 0.156 |
| Low dietary calcium intake (g/day) | **-** | 0.31 | 0.12–0.82 | **0.018** |
| Low BMI (kg/m^2^) | **-** | 0.91 | 0.79–1.06 | 0.220 |
| Low spine T-score | **-** | 0.63 | 0.40–0.98 | **0.041** |
| Low P1NP (µg/L) | **-** | 0.43 | 0.22–0.83 | **0.012** |
| hsCRP (mg/L) | **-** | 1.00 | 0.97–1.03 | 0.932 |
| Low plasma glucose | - | 1.35 | 0.76–2.41 | 0.305 |
| Non-fallers | - | 0.74 | 0.52–1.04 | 0.086 |
| No prior fracture | - | 1.29 | 0.85–1.96 | 0.229 |
| No cardio-metabolic disease | - | 0.68 | 0.47–0.97 | **0.036** |
| No cancer | - | 0.87 | 0.51–1.48 | 0.612 |
| No pulmonary conditions | - | 0.95 | 0.63–1.44 | 0.814 |
| No muscle weakness | - | 2.20 | 0.30–15.89 | 0.435 |

**Note:** P-values are derived from a time-dependent Cox proportional hazards regression model. *HR > 1 indicates increased risk; HR < 1 indicates reduced risk. Comorbidities are referenced against the absence or the lowest-risk group. Hazard ratios for continuous predictors represent the relative risk of joint replacement per 1‑unit decrease (BMI and ALM/h² per 1 kg/m² decrease; P1NP per 1 µg/L decrease; dietary intake per 1 g/day decrease). Reverse‑coding was applied to highlight protective associations. Variables were analysed in their original units and were not standardised*. *P-values < 0.05 are considered statistically significant.
*P-values between 0.05 and 0.10 are interpreted as marginally significant and marked with an asterisk (**).

**Abbreviations:** Hazard ratios (HRs); 95% confidence intervals (CIs); SES = Socioeconomic Status (based on IRSD: Index of relative socioeconomic disadvantage); BMI = Body mass index; ALM/h^2^ = Appendicular lean mass adjusted for height in meter square; BMD = Bone mineral density (measured at the spine); P1NP = Procollagen type 1 N-terminal propeptide; hsCRP = high-sensitivity C-reactive protein.

**Table S2**. Participant characteristics with missing values at baseline and follow-ups

| **Variable** | **Men** | | | **Women** | | |
| --- | --- | --- | --- | --- | --- | --- |
|  | **Baseline (*n =* 1,437)** | **5years (*n =* 911)** | **15years (*n =* 538)** | **Baseline (*n =* 1,446)** | **10year (*n =* 843)** | **15year (*n =* 647)** |
| Age (years) | 55.4 (38.8, 72.5) | 58.6 (44.8, 71.8) | 62.5 (51.3, 71.5) | 53.0 (36.9, 70.8) | 56.6 (45.0, 69.1) | 59.9 (49.4, 70.7) |
| sex |  |  |  |  |  |  |
| - Men | 1,437 (100.0%) | 911 (100.0%) | 538 (100.0%) | 0 (0.0%) | 0 (0.0%) | 0 (0.0%) |
| - Women | 0 (0.0%) | 0 (0.0%) | 0 (0.0%) | 1,446 (100.0%) | 843 (100.0%) | 647 (100.0%) |
| SES |  |  |  |  |  |  |
| - Low | 552 (38.4%) | 325 (35.7%) | 165 (31.1%) | 587 (40.6%) | 306 (36.3%) | 238 (40.4%) |
| - Medium | 257 (17.9%) | 166 (18.2%) | 125 (23.5%) | 306 (21.2%) | 164 (19.5%) | 142 (24.1%) |
| - High | 628 (43.7%) | 420 (46.1%) | 241 (45.4%) | 553 (38.2%) | 373 (44.2%) | 209 (35.5%) |
| - Missing | 0 | 0 | 7 | 0 | 0 | 58 |
| Smoking |  |  |  |  |  |  |
| - Never Smoke | 584 (40.6%) | 400 (43.9%) | 261 (48.5%) | 878 (60.7%) | 503 (59.7%) | 371 (57.3%) |
| - Past Smoker | 612 (42.6%) | 404 (44.3%) | 228 (42.4%) | 348 (24.1%) | 232 (27.5%) | 205 (31.7%) |
| - Current smoker | 241 (16.8%) | 107 (11.7%) | 49 (9.1%) | 220 (15.2%) | 108 (12.8%) | 71 (11.0%) |
| Alcohol intake |  |  |  |  |  |  |
| - Low | 774 (53.9%) | 478 (52.5%) | 444 (82.8%) | 1195 (82.9%) | 705 (83.6%) | 457 (73.8%) |
| - High | 661 (46.1%) | 433 (47.5%) | 92 (17.2%) | 247 (17.1%) | 138 (16.4%) | 162 (26.2%) |
| - Missing | 2 | 0 | 2 | 4 | 0 | 28 |
| Physical activity |  |  |  |  |  |  |
| - Active | 1,099 (76.6%) | 659 (72.9%) | 413 (77.1%) | 962 (66.7%) | 637 (76.5%) | 428 (69.5%) |
| - In active | 335 (23.4%) | 245 (27.1%) | 123 (22.9%) | 481 (33.3%) | 196 (23.5%) | 188 (30.5%) |
| - Missing | 3 | 7 | 2 | 3 | 10 | 31 |
| Dietary calcium intake (g/day) | 0.9 (0.7, 1.1) | 0.9 (0.7, 1.1) | 0.9 (0.6, 1.1) | 0.6 (0.4, 0.8) | 0.9 (0.6, 1.1) | 0.8 (0.6, 1.1) |
| - Missing | 98 | 37 | 5 | 9 | 34 | 45 |
| Weight (kg) | 82.6 (14.5) | 84.1 (14.5) | 84.9 (15.4) | 68.2 (14.3) | 72.6 (15.6) | 73.7 (15.3) |
| - Missing | 29 | 37 | 3 | 0 | 24 | 33 |
| Height (cm) | 174.9 (7.3) | 175.0 (7.2) | 174.8 (7.1) | 160.4 (6.8) | 161.3 (6.6) | 161.3 (6.3) |
| - Missing | 29 | 39 | 3 | 0 | 23 | 34 |
| BMI (kg/m^2^) | 27.0 (4.3) | 27.4 (4.2) | 27.8 (4.5) | 26.5 (5.4) | 28.0 (5.9) | 28.3 (5.8) |
| - Missing | 29 | 39 | 3 | 0 | 24 | 34 |
| ALM/h (kg/m^2^) | 8.6 (7.9, 9.2) | 8.4 (7.8, 9.1) | 8.4 (7.9, 9.1) | 6.5 (6.0, 7.1) | 6.7 (6.2, 7.2) | 6.6 (6.1, 7.1) |
| - Missing | 42 | 62 | 7 | 30 | 48 | 53 |
| Waist (cm) | 97.0 (11.4) | 97.8 (11.6) | 99.7 (13.3) | 84.7 (12.6) | 89.4 (13.7) | 89.6 (13.8) |
| - Missing | 58 | 50 | 4 | 34 | 44 | 27 |
| Hip T-score | −0.8 (1.1) | −1.0 (0.9) | −1.1 (0.9) | −0.9 (1.3) | −0.8 (1.3) | −0.9 (1.1) |
| - Missing | 42 | 67 | 11 | 6 | 45 | 56 |
| Spine T-score | 0.0 (1.4) | 0.0 (1.4) | 0.2 (1.4) | −0.6 (1.5) | −0.3 (1.4) | −0.3 (1.4) |
| - Missing | 41 | 60 | 22 | 3 | 41 | 56 |
| Systolic BP (mm/Hg) | 133.6 (16.6) | 139.8 (18.7) | 140.5 (17.4) | 125.5 (22.9) | 127.0 (17.8) | 133.4 (18.8) |
| - Missing | 101 | 106 | 3 | 53 | 72 | 35 |
| Diastolic BP (mm/Hg) | 83.2 (12.4) | 83.3 (12.1) | 79.7 (9.9) | 77.3 (12.8) | 77.2 (10.8) | 77.2 (10.9) |
| - Missing | 101 | 110 | 3 | 57 | 72 | 36 |
| Total Cholesterol (mmol/L) | 5.1 (1.0) | 5.2 (1.0) | 5.0 (1.0) | 5.5 (1.2) | 5.3 (1.0) | 5.3 (1.0) |
| - Missing | 333 | 106 | 105 | 159 | 108 | 90 |
| LDL (mmol/L) | 3.1 (0.8) | 3.2 (0.8) | 3.0 (0.9) | 3.0 (0.9) | 3.2 (0.9) | 3.2 (0.8) |
| - Missing | 348 | 118 | 110 | 122 | 110 | 92 |
| HDL (mmol/L) | 1.3 (0.3) | 1.3 (0.3) | 1.4 (0.3) | 1.2 (0.4) | 1.6 (0.3) | 1.6 (0.3) |
| - Missing | 333 | 106 | 105 | 123 | 108 | 90 |
| Triglycerides (mmol/L) | 1.6 (0.9) | 1.6 (0.9) | 1.5 (0.8) | 1.4 (0.9) | 1.3 (0.7) | 1.3 (0.7) |
| - Missing | 333 | 106 | 105 | 129 | 108 | 90 |
| hsCRP (mmol/L) | 1.8 (0.9, 3.4) | 1.7 (0.9, 3.2) | 1.4 (0.8, 2.5) | 2.1 (1.0, 4.4) | 1.9 (1.1, 4.8) | 1.9 (1.1, 4.5) |
| - Missing | 440 | 186 | 156 | 68 | 156 | 133 |
| CTx (ng/L) | 329.0 (235.0, 460.0) | 325.0 (234.0, 453.0) | 356.0 (269.0, 467.0) | 336.6 (213.5, 494.2) | 348.0 (247.0, 465.5) | 341.0 (244.0, 451.0) |
| - Missing | 364 | 134 | 105 | 74 | 108 | 90 |
| P1NP (µg/L) | 37.0 (27.0, 49.0) | 37.0 (28.0, 47.0) | 46.0 (37.0, 58.0) | 37.0 (26.0, 51.0) | 48.0 (36.0, 63.0) | 47.0 (37.0, 60.0) |
| - N-Miss |  |  |  |  |  |  |
| Plasma glucose | 5.4 (1.1) | 5.4 (1.2) | 5.4 (1.2) | 5.5 (1.2) | 5.3 (1.3) | 5.2 (1.2) |
| - N-Miss | 345 | 119 | 87 | 325 | 114 | 91 |
| Osteoarthritis | 109 (7.6%) | 68 (7.5%) | 116 (21.6%) | 166 (11.5%) | 187 (22.2%) | 156 (24.1%) |
| Prior fracture | 637 (44.3%) | 0 (0.0%) | 0 (0.0%) | 605 (41.8%) | 0 (0.0%) | 0 (0.0%) |
| Falls | 1,039 (72.6%) | 741 (82.3%) | 464 (86.6%) | 1,200 (83.4%) | 611 (73.6%) | 446 (71.8%) |
| - Missing | 6 | 11 | 2 | 8 | 13 | 26 |
| Diabetes | 107 (7.4%) | 76 (8.4%) | 59 (11.0%) | 73 (5.0%) | 71 (8.5%) | 50 (8.7%) |
| - Missing | 0 | 6 | 4 | 0 | 9 | 72 |
| Cardio metabolic conditions | 948 (66.0%) | 673 (73.9%) | 406 (75.5%) | 1,219 (84.3%) | 625 (74.1%) | 497 (76.8%) |
| Pulmonary conditions | 216 (15.0%) | 139 (15.3%) | 101 (18.8%) | 249 (17.2%) | 211 (25.0%) | 147 (22.7%) |
| Cancer | 185 (12.9%) | 133 (14.6%) | 140 (26.0%) | 121 (8.4%) | 149 (17.7%) | 115 (17.8%) |
| Muscle weakness | 23 (1.6%) | 11 (1.2%) | 11 (2.0%) | 15 (1.0%) | 8 (0.9%) | 6 (0.9%) |
| Calcium supplements | 45 (3.1%) | 60 (6.6%) | 5 (0.9%) | 123 (8.5%) | 145 (17.2%) | 149 (23.0%) |

**Note:** Values are presented as mean (standard deviation) for normally distributed continuous variables, median (IQR) for non-normally distributed continuous variables, and number (percentage) for categorical variables. P-values are based on: Independent t-tests for comparisons of means (continuous variables with normal distribution), Mann–Whitney *U* tests for non-normally distributed continuous variables (as applicable), and Chi-square tests (χ²) for categorical variables. Bold p-values indicate statistical significance at *p* < 0.05.

**Abbreviation:** JR = Joint replacement; SES = Socioeconomic status (based on IRSD = Index of relative socioeconomic disadvantage); BMI = Body mass index; ALM/h² = Appendicular lean mass adjusted for height squared; BMD = Bone mineral density at specific sites (s8 = spine, f0 = femoral neck), P1NP = procollagen type 1 N-terminal propeptide; hsCRP = high-sensitivity C-reactive protein; CTx = C-terminal telopeptide of type I collagen; HDL = high-density lipoprotein cholesterol, LDL = low-density lipoprotein cholesterol.
